# Diagnosis Between Chaos and Control: Affect and Hospital Clinicians' and Older Adult Patients' Narratives of Urinary Tract Infections

**DOI:** 10.3389/fsoc.2020.00057

**Published:** 2020-08-12

**Authors:** Paula M. Saukko, Emily Kate Rousham

**Affiliations:** ^1^School of Social Sciences and Humanities, Loughborough University, Loughborough, United Kingdom; ^2^School of Sport, Exercise, and Health Sciences, Loughborough University, Loughborough, United Kingdom

**Keywords:** affect, diagnosis, antibiotic prescribing, urinary tract infections, antimicrobial resistance, narrative analysis

## Abstract

Research has observed that older adults are frequently overdiagnosed with urinary tract infection (UTI) and unnecessarily prescribed antibiotics in hospitals. In this article we explore the overlooked affective dimension of experiences of diagnosis and prescribing. Drawing on interviews with doctors, nurses and older adult patients (*n* = 41) on UTI diagnosis in two UK hospitals and Arthur Frank's work on illness narratives we identified two affective ways of experiencing diagnosis. Some clinicians and older adult patients articulated *chaos* narratives about being overwhelmed by contradictory evidence and events, doubting the repeated UTI diagnoses and courses of antibiotics but being unable to do anything about their concerns. Other clinicians and patients articulated *control* narratives about UTIs being frequently diagnosed and antibiotics prescribed to restore patients' health, echoing certainty and security, even if the processes described typically did not follow current guidance. We contend that analyzing the affective dimension offers conceptual insights that push forward sociological discussions on diagnosis as reflective or dogmatic in the context of the contradiction between acute care and chronic illnesses of old age. Our findings contribute practical ideas of why overdiagnosis and overprescribing happen in hospitals and complicate notions of patients pressuring for antibiotics. We also present methodological suggestions for analyzing *how* participants tell about their experiences in order to explore the typically not directly spoken affective dimension that influences thoughts and actions about diagnosis.

## Introduction

Older adults frequently have bacteria in their urine without symptoms (asymptomatic bacteriuria), which should not be treated with antibiotics. Clinical guidance in the UK recommends that UTIs should be primarily diagnosed based on symptoms, such as pain when passing water, rather than presence of bacteria in urine identified by diagnostic tests, such as point-of-care urinary dipsticks or bacterial cultures (Scottish Intercollegiate Guidelines Network, 2012; Public Health England, 2018). However, international research has observed that this guidance is frequently not adhered to in hospitals (Pallin et al., 2014; Lee et al., 2015; Eyer et al., 2016) or care homes (Chambers et al., 2019) contributing to antimicrobial resistance (AMR).

Qualitative research has found that junior doctors' overdiagnosis of UTIs and unnecessary antibiotic prescribing in hospitals is driven by overreliance on laboratory results, risk aversion, difficulties in interpreting symptoms and perceived pressure from peers, patients, and families (Eyer et al., 2016). Research on antibiotic prescribing in general has found that doctors typically focus on the immediate risk of infection for their individual patients rather than the communal, future risk of AMR (Broom et al., 2014; Krockow et al., 2019). It has also been observed that antibiotics are prescribed to appease other staff, patients, and families in hospitals (Lewis and Tully, 2009; Charani et al., 2013), that there are problems in interaction between different staff, patients and clinical domains (Skodvin et al., 2017; Saukko et al., 2019), junior doctors are confused by contradictory advice (Mattick et al., 2014; Kajamaa et al., 2019) and that professional identities (Broom et al., 2016) and “off label” local cultures fuel prescribing (Caronia and Saglietti, 2017).

Previous studies resonate with medical sociological work on diagnosis, such as junior doctors' reflections on uncertainty and whether it relates to their lack of knowledge or uncertainties of medical knowledge itself (Fox, 1980), how junior doctors' decisions may not only be characterized by uncertainty but also by unreflective, learnt stock responses (Atkinson, 1984), different ways of using evidence by doctors (Timmermans and Angell, 2001), the contradictions between focusing on disease manifesting itself in pathology, such as laboratory results, and illness articulated through patients' descriptions of symptoms (Armstrong, 2011) and how clinicians do not necessarily follow guidelines but, for example, rely on their intuition and consider organizational issues (Carthey et al., 2011; Johannessen, 2017). The empirical and conceptual research illustrate different factors and approaches at play when clinicians encounter an ambiguous situation, such as a suspected UTI in an older adult. Our qualitative interviews with doctors, nurses and older adult patients in hospitals corroborated many of the previous observations. However, we contend that previous research has not considered the affective feelings (Massumi, 1995; Seigworth and Gregg, 2010), which underpin experiences of diagnosis and antibiotic prescribing.

The first author initially noticed that our interviews with clinicians and older adult patients gave off a sense of being overwhelmed by contradictory evidence, repeated UTI diagnosis, and antibiotics, having doubts about the procedures but being unable to act on the concerns. Other participants told about diagnosis and antibiotic use with a sense of certainty and security, even if the processes described did not follow current guidance. Participants did not directly tell about these affective experiences, such as saying that they were bewildered. Rather, the form of narratives in the interviews (*how* stories were told) brought to the fore the sense of being out of or in control. In making sense of these stories we drew on Arthur Frank's classic work on types of patients' illness narratives (Frank, 1995), arguing that UTI diagnosis and antibiotic prescribing for older adults could be experienced in terms of *chaos* and/or *control*.

We contend that analyzing the affective dimension offers conceptual insights that push forward sociological discussions on diagnosis as reflective or dogmatic in the context of the contradiction between acute care and chronic illnesses of old age. Our findings also contribute practical ideas of why overdiagnosis and overprescribing happen in hospitals and complicate notions of patients pressuring for antibiotics. We also present methodological suggestions for analyzing *how* participants tell about their experiences in order to explore the typically not directly spoken affective dimension that influences thoughts and actions about diagnosis.

## Diagnosis and Affect

Classical sociological work on diagnosis in hospitals has focussed on junior doctors' experiences of reflecting on uncertainties of medical knowledge or evidence or relying on routine or dogmatic stock responses to clinical situations (Fox, 1980; Atkinson, 1984; Timmermans and Angell, 2001). Antibiotic prescribing decisions have been found to lean toward the dogmatic side of the equation and doctors have been observed to overprescribe focusing on the risk of infection (Broom et al., 2014). Nurses have been found to push for antibiotics seeing themselves as advocates for patients (Broom et al., 2016), and nurses have also been observed to follow internalized “mindlines” rather than guidelines in triage decisions (Johannessen, 2017), highlighting the fine balance between too strict following of either guidance or intuition.

Sociologists typically consider critical reflection more sensitive to the multi-faceted nature of medical decisions and patient experiences than unquestioning following of routines or guidance (Timmermans and Angell, 2001). However, reflection is a rational, solution-driven activity, even if it has been acknowledged that it can be accompanied by feelings of self-doubt (Fox, 1980). In our research we noticed that clinicians and older adult patients could have doubts about diagnosis or prescribing decisions, but these doubts did not necessarily lead to a different line of action but to a sense of unease. At the same time routine practices potentially leading to overdiagnosis were described with a sense of certainty. We contend that these observations point to a neglected affective dimension of diagnosis and prescribing that importantly influences decisions and renders them intelligible.

To capture the above mentioned feelings we use the term affect rather than emotion. It is not our intention to participate in debates about the currently fashionable interest in affect in social sciences (Wetherell, 2015). In psychology affect usually refers to a visceral layer of experience, whereas emotions are understood to be cognitively recognized states, such as sorrow or joy (Russell, 2003). We take the lead from post-structuralist work that sees affect as feelings that may or may not be cognitively and verbally articulated and that emerge from relations between people, events and things (Deleuze and Guattari, 1987; Massumi, 1995; Gregg and Seigworth, 2010).

The important thing here is that the sense of unease or security were not necessarily directly verbalized in the interviews but conveyed through the way in which events were described. To gauge this affective dimension we resorted to narrative analysis, which has been used in medical sociology and health services research to bring to the fore the embodied and emotional dimension of experience often silenced in biomedical research. Early narrative research often focused on patients' emotional experiences (Hurwitz et al., 2008). Recent work, more directly pertinent to our research, has analyzed UK junior doctors' narratives on regulation of emotion (Lundin et al., 2018) and preparedness for practice (Monrouxe et al., 2018). Closest to our topic there is also research on junior doctors' narratives of antibiotic prescribing about feelings of being unsupported or given contradictory advice (Mattick et al., 2014; Kajamaa et al., 2019) as well as on nurses' narratives on their experiences of speaking up about safety concerns (Law and Chan, 2015).

Narrative analyses have drawn attention to clinicians' and patients' uncomfortable experiences, such as a sense of powerlessness, which influence clinical practice. However, the analyses mostly focus on the content of the narratives (*what* is being told) rather than the form (*how* is it being told) (Chatman, 1978). Form is the less obvious dimension of narrative, but it is saturated with meaning; for example, the classical research article, using a passive voice and a detached description, communicates authority, and objectivity. The scientific article illustrates how narrative forms frequently follow normative cultural scripts. Drawing on structuralism and phenomenology Frank (1995) argues that forms of illness narratives articulate both social scripts and not necessarily conscious embodied and emotional ways of relating to the world.

Frank (1995) delineates basic illness narratives, of which we will discuss two that resonated with our study. The first, restitution narrative rehashes the classical normative script of a patient falling ill and biomedicine heroically restoring his/her health; the order of events is linear, orderly and achieves closure. The second, chaos narrative is disorderly, events occur out of sequence and repetitively, the illness overwhelms the experience and there is no resolution to the situation. As indicated earlier the forms of narrative identified by Frank repeated in our interviews, highlighting the affective dimension of overdiagnosis and overprescribing that pushes forward discussions on reflexivity, dogmatism, adherence to guidance and why overdiagnosis happens.

## Methods

Our study explored processes of diagnosing and treating UTIs in older adults in two hospitals in the UK Midlands. After obtaining ethical approval from Healthcare Research Authority (IRAS 202255) we put posters about our research on the walls and published information on relevant staff e-newsletters. Afterwards a research nurses visited wards, handing out information packs and invitations to participate to: (i) healthcare staff involved in diagnosing UTIs in older adults and (ii) older adult patients (>70 year olds) who had been diagnosed with a UTI during their current hospitalization. Staff and patients who expressed an interest in taking part were contacted by an experienced qualitative researcher who arranged for an interview.

We recruited a total of 41 participants, including 27 healthcare staff, comprising of 13 nurses, 9 doctors, 3 healthcare assistants and 2 microbiologists, and 14 older adult patients. Most of the doctors (7) were junior doctors, who mostly perform initial UTI diagnosis. The doctors were recruited from both acute (A&E) and subacute wards, the patients and nursing staff were recruited from subacute wards, including older adults, orthopedics, stroke, and rehabilitation. The average age of the patients was 81 (range between 71 and 89) and they were all assessed by the research nurse to be cognitively capable of giving informed consent. Staff were asked to describe their job role, how diagnosing UTIs in older adults featured in their work, how they went about the diagnosis, their role in prescribing, perspectives on recovery, and any concerns they may have. Patients were asked to tell about being diagnosed with a UTI, experiences with treatment, prior experiences with UTIs and other health related issues and any concerns they may have. All bar one interviews with staff took place in a private room in the hospital, one in a clinician's home. Six patients were interviewed in the hospital, seven at home after discharge and one, who lived in another area, was interviewed by phone. The average length of the interviews was 24 min, ranging between 12 and 43 min; some interviews were short due to older adult patients in the hospital being frail and getting tired and some clinicians being busy. Most of the interviews were thus not in depth but short conversations carried out with clinicians over a break or with convalescing patients, which did not necessarily gauge significant amounts of background information but could capture the experiences of diagnosis and/or illness in the hospital environment.

The interviews were transcribed in verbatim. We first analyzed the interviews using the constant comparative method (Glaser, 1965) and observed that they featured two broad themes of “control” and “chaos.” Following the principles of abductive analysis (Timmermans and Tavory, 2012) we brought Frank's (1995) work to bear on the material, reading back and forth between literature and material. The two broad themes were coded into subthemes, based on key moments in diagnosis e.g., symptom recognition, facilitated by the use of NVivo 10 qualitative software. A selection of transcripts was read by all members of the research team, and the coding scheme was developed building consensus within the team, including two clinical members who are not authors of this article. A previous article focused on the subthemes, discussing the different staff groups' and older adult patients' divergent understandings of the key stages of diagnosis leading to problems in “translation” (Saukko et al., 2019).

This article focuses on the broader themes of chaos and control, defined as affective states, characterized by (i) sense of being out of control, experience of contradictory events/perspectives, doubts about the righteousness of actions, and an inability to act on concerns, and (ii) sense of being in control, experience of orderly series of events, sense of “doing the right thing,” and problems being solved. The sense of chaos and control was articulated through how the narratives were told. To systematically analyse these narrative forms we used insights from Frank (1995) and other work on narrative analysis (Chatman, 1978; Riessman Kohler, 1993; Stephens and Breheny, 2013) to discern three key aspects of the interviews: (i) how the teller positioned him or herself in relation to his/her and others' actions, (ii) the coherence of the sequence of events and perspectives, and (iii) whether the story achieved closure or resolution. It should be noted that individual interviews could be dominated by either chaos or control narrative but interviews often shifted between the two.

In what follows we will first present the general characteristics of our material, then discuss the chaos narratives featuring in the interviews, and move on to discuss control narratives.

## Findings

The overall feature of our material was that the descriptions of processes of diagnosing UTIs and prescribing antibiotics for older adults did not typically follow the ideal proscriptions of clinical guidelines (Scottish Intercollegiate Guidelines Network, 2012), which was also corroborated by our parallel quantitative case series review of patient records (Rousham et al., 2019). The sense of chaos vis a vis the diagnostic process was most prominent in the junior doctors' and older adult patients' interviews and less common among nursing staff; the sense of control and certainty was most common in nurses' interviews and was less common in doctors' and patients accounts. In what follows we will present our findings through illustrative cases from doctors, patients, and nurses to capture the experiences of different groups. The cases have been selected to represent both intense and typical (often less clear) cases (Patton, 2015) seeking to do justice to the richness and nuances of the material. The names used are pseudonyms and some details have been modified to protect anonymity.

### Diagnosis as Chaos

#### “Maybe It's a UTI”

First example of a chaos narrative is the description by Anthony, a junior doctor, of a typical situation of encountering UTIs:

**So, when do you encounter UTIs—?**
It crops up because I see quite a few patients in acute medical unit. They come in just feeling actually unwell and they can't tell you what's wrong with them as they're too confused sometimes, and then you have to somewhat think, “Oh, maybe this is an infection causing delirium and maybe it's UTI,” which is one of the most common causes anyways. … Maybe the family says, “Oh, this patient she's been having like foul-smelling urine and pain, so maybe it's UTI.” … The over 65 group, they're confused, they don't know where they are. They can't really explain what's going on. They sound like they have dysuria [pain when urinating] or maybe not, not too sure, and yeah, just think about UTI and then get all the investigations done and we think about and hope it is the UTI.

Anthony switches between the pronouns “I,” the impersonal “you,” use of passive voice and in the end evoking the collegial “we,” fluctuating between owning and distancing himself from his actions and having and lacking agency. The older adult patients are referred to impersonally as “they,” more as objects to be observed rather than subjects to be engaged with reference to vague illness (actually unwell), potential UTI symptoms (sound like they have dysuria, maybe not, not too sure), and cognitive impairment (cannot say what's wrong with them, they don't know where they are, what's going on). Anthony's narrative describes his unsure (“you gotta somewhat think”) attempt to match the symptoms to a textbook diagnosis (maybe infection causing delirium) to justifying his actions with reference to ostensibly factual common sense (it is one of the most common causes). Characteristic of chaos narrative many contradictory events happen all at once (patient is unwell, families tell they may have dysuria, patients don't know where they are, investigations are done) and the narrative does not proceed in an orderly fashion and achieve a resolution or closure. The felicitous nature of diagnosis is left unclear with a query “maybe it's UTI” and “hope it's UTI” left hanging in the air, reflecting Anthony's doubts about the diagnostic process.

Anthony's interview could be read from the point of view of content of the narrative (what is told), corroborating that junior doctors find interpreting symptoms of UTI difficult (Eyer et al., 2016). The form of Anthony's interview, however, opens another affective and not directly verbalized perspective on the experience of UTI diagnosis. Similar to Mattick et al. (2014) analysis of junior doctors' antibiotic prescribing narratives, Anthony shifts between a position of knowing and not knowing in his interview. However, Anthony's narrative mainly communicates him being tugged and pulled into contradictory directions by different clues about older adult patients' symptoms. In an earlier part of the interview Anthony described similar series of events in interpreting inconclusive and contradictory diagnostic tests. Overall, his narrative gives off a sense of being overwhelmed by contradictory evidence and of being acutely aware that the default position of UTI diagnosis (“maybe/hope it's a UTI”) is not necessarily the right one whilst being unable to do anything about it.

Catriona is a junior doctor working in a rehabilitation ward. At the start of the interview she describes processes of diagnosing UTIs in older adults echoing confidence and control, underlining how senior consultants had instructed her not to pay too much attention to urinary dipstick results. However, when describing processes of collecting urine samples, the form of her narrative changes:

**Could you tell me about the urine collection … is it tricky with the older adults?**
I'd say yes. A lot of the time I'll optimistically ask for a specimen and then I'll be told actually they're incontinent. Then when they're incontinent, then I will say can we dip the [continence] pad. Which probably isn't best practise, but it's the best we can do given the circumstances. …
**How do you interpret the results against the fact that they've been taken from a pad?**
… If I know it's been taken from the pad, I don't think it really changes the way that I would interpret it to be honest. I think I would interpret it the – I think I would – bear in mind it's been taken from the pad and probably it's not going to be as accurate as we would like it to be, because it's not the way it's intended to be used. I guess sometimes you have to make do with what you've got.

In this excerpt Catriona mainly uses the first person pronoun “I” articulating herself as the agent of actions. However, at the end of both paragraphs, where doubts about the righteousness of the procedure appear, she shifts into a collective “we” and passive voice, distancing herself from the actions and her own agency. In the second paragraph Catriona's narrative takes the form of thinking out loud which line of action she would be taking (“if I know,” “I don't think,” “I would … to be honest,” “I think I would”), shifting between positions and articulating hesitancy. The narrative resembles bouncing off the walls in a blind alley until it becomes clear there is no way out or resolution, and Catriona concludes that diagnosing UTIs based on dipsticking urine taken from a continence pad is something one “has to make do with” even though she is acutely aware of this not being the ideal course of action.

The content of Catriona's interview corroborates qualitative and quantitative observations from hospitals care homes that urine samples are not often collected midstream (Schweizer et al., 2005; Pallin et al., 2014). However, the focus on narrative form and affect illustrates how a junior doctor's shifting around different scenarios in relation to urine collection and interpretation of results, concluding it is impossible to do it right.

The forms of junior doctors' narratives of diagnosing UTIs in older adults highlight the affective experience of being overwhelmed, faced with complex and/or impossible to solve situations, being ambivalent about the righteousness of one's actions and unable to do anything about it. The interviews articulate elements of uncertainty (Fox, 1980), routine practices (Atkinson, 1984), erring on the side of overinterpreting risk (Broom et al., 2014) and not adhering to guidelines (Carthey et al., 2011). However, these works on clinicians and diagnosis do not capture the experience of repeated and seriously doubted actions, which open another perspective on how overdiagnosis of UTIs in older adults and overprescribing of antibiotics happens in hospitals.

#### “Tablets for Something”

Older adult patients' interviews often articulated a sense of chaos amidst their frequently complex care. For example, Elena is in her late eighties, has heart failure, has recently been diagnosed with breast cancer and relates she has been repeatedly diagnosed with UTIs and describes how she was hospitalized during the latest episode of UTIs:

I couldn't breathe anymore, and I pressed that button [home alarm] … and they sent the ambulance … and then they said I have to go to hospital. … And when I came there – I already had a water infection there, and they gave me something for it. And then they gave me something to breathe, you know, some tablets, antibiotics or something…. Then the ambulance brought me back again. … I got different tablets for two weeks from the hospital … Anyway, I got different tablets, but they took the water tablets away... And I don't know why … But then afterwards it might be they took it away and then maybe they'll bring it back again, we don't know. Because I've got a kidney infection as well …
**Oh, so did the water infection go to your kidneys this time around?**
No, the water infection, I had an x-ray in the hospital and they said I had water on the lungs. And that's why they gave me something for it, and then I think antibiotics.

Elena uses the first-person pronoun “I,” relating her experience of events, and refers to clinicians impersonally as “they.” Characteristic of chaos narrative Elena's account of events is repetitive, referring to “tablets,” which are given, taken away and possibly brought back. Some of the tablets are possibly antibiotics (“or something”), some of them are for UTIs, others for other ailments, such as water in the lungs. The sequence of events in the narrative is blurred, many events happen all at once, it becomes difficult both for Elena and the reader/listener to make sense of what happens and in what order. There are multiple illnesses (breathing, UTIs, heart, lungs, kidney infection) and multiple treatments involved. All the events, illnesses, and treatments create a jumbled-up narrative and a strong sense of being overwhelmed amidst too many health-related things going on. Elena, the protagonist, is clearly not in control of the events, which are mainly driven by the impersonal clinicians, referred to as “they.”

Elena's narrative illustrates the affective experience of being at the mercy of medical interventions happening to her. Her narrative exemplifies how co-morbidities or multiple illnesses and treatments of old age intermesh with the UTI diagnosis making them all blend into a chaotic, anxiety-riddled experience. Research on clinicians has reported that they perceive patients to “pressure” for antibiotics (Eyer et al., 2016). Elena's narrative did not indicate pressure, rather her narrative communicates a sense of being overwhelmed with her repeated diagnoses and medications. Elena's narrative is also indirectly critical of clinicians, who are referred to as an anonymous force (“they”), whose actions she observes without being able to fully understand them and not being given explanations.

Philip is in his early eighties, had experienced balance problems and several falls, which had been the original reason for his admission. He has been diagnosed with a UTI, at the time of the interview his hospitalization had prolonged, his UTI is unresolved and his balance problems continue to be investigated. He describes antibiotic prescribing for his UTI as follows:

**How did they treat you for this water infection?**
By medicine I think, tablets and that, trying to—
**So it was tablets?**
Yes. I'm on a lot of tablets, believe me (laughs).
**Okay, just for this or just in general?**
Yes, in general. I should be rattling by now all that I've had (laughs).
**What kind of medicines do you take?**
It's all tablets.
**What's that for?**
Don't ask me, my dear. I couldn't tell you. I know there was some – when I first came in there was water tablets and then I [was put on IV antibiotics].

Philip tells his story in first person, from his perspective, but refers to “tablets” in passive voice as “it's” and “there was” indicating he is not actively taking medications but they are being administered to him. Similar to Elena's narrative, the prescribing of a “lot” of unspecified medications for various, unclear reasons repeat. Philip is not in control of events, he does not know exactly what medications he has been given. The narrative does not achieve a resolution, Philip's hospitalization, antibiotics/tablets, UTI, and balance problems all remain on-going. However, Philip articulates less urgent worry and dissatisfaction with care than Elena, he frames himself as having accepted a passive role and intersperses the interview with humor throughout even though he is frail and has to make an effort to speak during the interview.

Hospitalized older adults' experiences of UTI diagnosis have not, to our knowledge, been studied before. Clinicians have been observed to perceive patients and families to pressure for antibiotics for UTIs in hospitals (Eyer et al., 2016) and care homes (Chambers et al., 2019). However, rather than pressure for antibiotics our findings resonate with older adult experiences of taking multiple medicines (polypharmacy) in community has been found contradictory, often accepting of the necessity of medications but also having concerns and not understanding or being explained the purpose of different medications (Moen et al., 2009; Clyne et al., 2017). The narratives of our older adult patient participants highlight an affective experience of repeated UTI diagnoses, other illnesses, and medications, creating an undistinguishable amalgam of medical interventions which patients do not fully comprehend interlaced with greater or lesser amounts of worry.

#### Antibiotics and Dipsticks Again

An example of a nurse narrative structured by chaos is Elias' interview. He works on an older adults' ward and discusses how older adult patients often had repeated UTI diagnoses and courses of antibiotics, returning to the ward and the bacteria becoming resistant to antibiotics:

**So, you see the same patients with recurrent UTIs?**
Usually, yeah.
**So how common is that, that they … come again?**
As I've said, because they become resistant to treatment—Yeah. So, in the acute stage, again they will give strong antibiotics, like to which antibiotic they are responding. Some of them are still responsive, but it kind of takes a while before they get better. And then maybe, I could say, I think, they kind of become a carrier— What do they call that? They are harbouring the bacteria, but they are not symptomatic … So, any time they can flare up, if their body could not – if the immune system is low, is down. So, it can flare up again, then treat again with the kind of strong antibiotics and then come back again.

The interview is characterized by repetitive recurrence, characteristic of the chaos narrative of older adult patients being prescribed “strong antibiotics,” how it takes patients longer to recover, until the bacteria becomes resistant to antibiotics and the patient is treated with another course of “strong antibiotics” and, yet again, returns to the hospital. The narrative has a strong sense of powerlessness, which is articulated through mainly use of passive tense, treatment decisions being made by an impersonal institutional agent, interspersed with occasional use of first person pronoun to indicate Elias' hesitant critical own view with “I think” “I could say.”

Elias's narrative goes against observations of clinicians' behavior vis a vis inappropriate antibiotic prescribing in hospitals. It has been noted that doctors consider antimicrobial resistance an abstract threat in the future, whereas the risk of infection for their patients is more immediate and concrete (Broom et al., 2014). In Elias's narrative the risk of antimicrobial resistance is concrete, affecting nursing staff on the ground who witness bacteria colonizing older adult patients becoming resistant and returning to the ward with UTI diagnosis. Nurses have been observed to indirectly push for antibiotics, in the interest of the patients (Broom et al., 2016). However, Elias's narrative illustrated the difficult position of nurses that witness recurrent, potentially unnecessary prescribing of antibiotics without being able to do anything due to their position in the hospital hierarchy, which has been observed in narrative research on safety issues generally (Law and Chan, 2015). The narrative reflects the affective experience relentlessly repetitive antibiotic prescribing, powerlessness and urgency, almost despair.

Most interviews with nursing staff did not contain intense or prominent chaos narratives. The interviews often had parts where a confusing aspect of diagnosis were discussed but then the interview restored normality or control, as in the interview with Sonia, a healthcare assistant in an older adults ward:

I think because we get a lot more dementia patients now, it can be very tricky. There are a lot more patients that have issues with ulcers and things and there are fluid balance charts and things like that, you know, fluid restriction because they get a lot water in the legs and things like that and that could cause you to wee a lot. So if they've got that, as well as the weeing a lot, and they're on a fluid restriction, sometimes it can be difficult. But the actual test isn't so difficult. So, you know, if you're suspicious, it's just a urine dip and you roughly get a good idea if something else is going on.

In the excerpt Sonia discusses patient care mostly in passive tense (“there are,” “it can be”) not implicating herself in the actions directly. She discusses various other conditions and symptoms typical in older adults (ulcers, fluid balance charts, fluid restriction, weeing a lot) that could confound UTI diagnosis and affect urination and (de)hydration. However, even if Sonia's interview has aspects of chaos narrative in terms of introducing contradictory series of events, it achieves closure through using a urinary dipstick.

The nurses' experiences of being overwhelmed by contradictory events is similar to those of doctors. However, nurses' narratives also illustrate the affective dimension of their powerlessness in the organizational structure as well as how the use of urinary dipsticks, which goes against clinical guidance, becomes a means to resolve a confounding and contradictory situation.

### Narratives of Control

Whereas, the chaos narratives were characterized by a sense of events spinning out of control, in the control narratives the narrators present themselves as in charge of the processes of UTI diagnosis.

#### “Every Patient Has a Urinalysis”

Anette is a senior nurse in an older adults' ward and describes her job as “making sure that the care that's being delivered is at a high standard, and that we're meeting all the measurements and the targets.” So, she presents herself as in control of not only her work but of the overall care in the ward. She describes UTI diagnosis in older adults in terms of a routine sequence of procedures followed:

Well every patient that comes onto the ward has a urinalysis, so on admission to the ward they'll have a urinalysis done. …
**You mentioned urinalysis, how is that done?**
Through a dipstick. Yeah, so their urine is dipsticked on arrival to the ward, and then obviously if they've got blood, or leukocytes then obviously that's escalated to the medics and then we automatically send a specimen away to the lab. … If it's positive then obviously a specimen is sent off, medics are involved; medics usually don't prescribe until they've actually got the result back from the microbiologist. But if the patient is symptomatic, got a temperature or just feeling generally unwell then they will prescribe, I think it's three or five days of trimethoprim. … because they've obviously, urosepsis and things like that, we've got to be careful of.

Anette mainly uses the passive voice, her narrative position observes events from a distanced, managerial perspective, different actors of the process are referred to in terms of professional groups as “staff,” “patients,” and “medics.” She occasionally identifies herself with the nursing staff as “we,” referring to sending specimens to the laboratory, changing to a more encompassing “we,” including doctors, when referring to the need to be careful about sepsis. Anette's narrative presents diagnosis of UTIs in older adults proceeding in an orderly sequence; every patient is dipsticked on admission; if the dipstick is positive, samples are sent to the laboratory and if the patient is symptomatic or generally unwell, antibiotics are prescribed, to avoid sepsis. Anette's narrative gives off a sense of control and of doing the right thing. The diagnostic procedures described, however, are largely at odds with clinical guidelines, which recommend against the use of dipsticks in diagnosing older adults, on admission or otherwise.

Anette's orderly narrative with a resolution (Chatman, 1978) articulates an embodied, affective sense of control and order. The narrative illustrates how patterned processes of diagnosis, which conform to cultural scripts of biomedicine curing disease, lend them affective force.

The sense of control or straightforwardness was also echoed in descriptions of UTI symptoms, as illustrated by an account by Ellie, a nurse in a ward for older adults:

**What alerts you to a UTI?**
Okay, it may be that the patient is showing signs of confusion and they're not normally confused, so that would alert me that they may have a UTI. I'd want to rule that out first. So I'd get a urine sample, obs them, see if they've got a temperature, see if they're tachycardic, anything else away from the baseline, but get some urine for urinalysis, dip it and see and send off a specimen if they have – if it's a positive dip, so if they've got leucocytes, nitrates, protein, blood, anything out of the normal really.

In this excerpt Ellie uses first person pronoun, indicating her sense of agency. The answer does not echo as strong control as Anette's reply but—even if Ellie elsewhere in the interview acknowledges that UTI diagnosis in older adults may be “tricky” because of “comorbidities”—it presents identification of symptoms as a fairly simple, linear process of looking for confusion, taking temperature, dipping the urine, and sending a urine sample to the lab if the dip is positive.

Previous research has found nurses to push for antibiotics (Broom et al., 2016) and that doctors' may perceive nurses to pressure for antibiotic and prescribe to appease them (Charani et al., 2013; Eyer et al., 2016). The interviews by Anette and Ellie do not necessarily indicate active pushing; rather the descriptions of orderly UTI diagnosis, when they could lead to overdiagnosis, have a sense of being self-evident. The nurses' descriptions are similar to the junior doctors' “stock responses” observed by Atkinson (1984) but also highlight the affective confidence and certainty afforded by adhering to old practices that lend them force.

#### “Then I Was Treated”

Most older adult patient narratives had elements of chaos even if they also articulate a sense of control in part of the interview.

Joanne is in her seventies, and her interview is an unusually clear case of control narrative, Similar to Elena, Joanne has recurrent UTIs, which she relates to urine retention or “when I have a wee my bladder doesn't empty completely.” She describes her hospitalization matter of factly of not being able to walk, being taken to hospital by ambulance and being treated for a UTI. Reflecting on the repeated UTIs, together with her husband in the room, she stated that she was not perturbed by recurrent UTIs:

**Are the water infections a big bother?**
Not really.
**So they kind of come—**
And go.
**… How do you find the tablets?**
All right.
*She gets antibiotics*.
Yeah, antibiotics.

Joanne's replies to questions are short, and she completes or corroborates the interjections by the interviewer and the husband. Joanne maintains that she is not overly concerned about the repeated UTI diagnoses and in the curd answers here as well as elsewhere in the interview she presents antibiotics as solving the problem. Joanne's narrative does not necessarily frame herself as in control, rather the narrative achieves resolution and UTIs are being controlled by antibiotics administered by healthcare professionals in recurrent, predictable manner, restoring normalcy in Joanne's life who comes across as a willing object of treatment.

Joanne's narrative suggests that she might be a patient who could expect antibiotics for her suspected recurring UTIs as suggested by clinician interviews (Eyer et al., 2016). However, Joanne's interview also illustrates the affective force of control and security afforded by the restitution narrative (Frank, 1995) that promises that biomedicine cures disease and restores health amidst repeated illnesses in old age.

Most, older adult patients' interviews mixed elements of chaos and control. For example, Alison, in her seventies, had undergone a rectal operation in the hospital when diagnosed with a UTI; after discharge she was diagnosed with a UTI again by her GP based on laboratory results, as she tells:

They must have given me antibiotics. Yes, it did, but it [water infection] came back for some reason, but I'm not surprised because, excuse me saying it, I was on the toilet most of the time.
Second time it [the urine] was cloudy, because I didn't see the first time because they tested it, but I certainly saw it the second time.
**Yes, okay. So, it didn't hurt and you didn't run a temperature or anything like that?**
No, not at all.
**… Okay. So, when you had it [the urine] tested the second time, was that with your GP?**
Yes.
**Okay, so did they just suggest that you might want to have it done again or—?**
Oh, yes, absolutely, and that's when I had it done again after taking antibiotics and it cleared.

Alison shifts between first person pronoun describing her actions (“I was on the toilet,” “I saw the [urine]”) and passive voice when describing diagnostic tests, treatments and the disease (“tested it,” “treated it,” “it came back”) indicating her passivity in relation to medical interventions and agency in terms of observing them. In Allison account antibiotics are given, infection comes back, urine is tested, urine is cloudy, there is no pain and urine is tested again by GP, all having elements of chaos narrative where things happen repeatedly in a somewhat uncertain order and not always making sense. However, the narrative achieves closure as eventually antibiotics clear the infection, restoring, if not health (as rectal problems persist), at least curing infection.

The patients' control narratives highlight the affective sense of resolution afforded by antibiotics in the context of repeated chronic diseases of old age. However, as the two narratives illustrate, the intensity of this affect varies, Joanne clutching to the restorative powers of antibiotics, whereas for Alison treatment by antibiotics is simply accepted, highlighting that even if patients consider antibiotics a resolution they are not necessarily invested in them in equal measure.

#### Tradition and Counter-Tradition

The doctors' interviews often combined elements of control narrative and chaos narratives. Typically, doctors discussed UTI diagnosis in terms of being in charge of an uncomplicated process at some point in the interview but shifted into voicing doubts and contradictions at another point.

Anya is a junior doctor, who at the time of interview works in the rehabilitation ward but had recently worked in A&E. Much of her interview conforms to the control narrative. In answering one of the first questions on typical situations where UTI was diagnosed, Anya offers an orderly account:

Typical situations will be patients coming with some sort of falls or infections and we do an in-depth to see if – we either think of chest or urinary, those are the causes most of the time. … So, once the patient comes up to acute medicine we do a urine dip and if it's positive for nitrites, leukocytes, we generally ask them to send it to the lab for culture and see if it's growing anything. If there are any signs of sepsis or white cell count is too high, CRP is high, patient is not clinically well, we start the patient on antibiotics anyway.

Anya uses the pronoun “we,” evoking a narrative of a common collegial practice among (junior) doctors, rather than an individual one. She describes the process of UTI diagnosis in older adults as unproblematic, proceeding from identifying signs (falls), testing the urine with a urinary dipstick and prescribing antibiotics, if patients are unwell or there are any suspicion of sepsis. Anya's description follows the familiar sequence of events, repeating in many interviews with clinicians, of focusing on vague signs (falls), using urinary dipsticks for diagnosis, and prescribing antibiotics, when older adult patients are unwell. This sequence of events does not necessarily adhere to current clinical guidance. However, the coherent order of events and the closure brought to the narrative by antibiotics communicate a sense of control and certainty, Anya's story lets on that she is doing the right thing.

Stephen's interview is unusual in that even though it echoes control it does not repeat the restitution narrative (Frank, 1995), whereby curing disease brings closure to the story. Stephen is a senior consultant in a stroke ward and underlines throughout the interview that he does not diagnose UTIs in older adults based on identifying bacteria in urine:

**What kind of alerts you to a UTI?**
Preferably symptoms, new onset pain, discomfort and passing urine, passing urine more often, plus or minus fever. Then you might want to back it up with urine culture, but I wouldn't primarily diagnose it just on the basis of an E. coli urine culture coming back again because it's not necessarily right. It doesn't necessarily mean anything, to be honest. So preferably symptoms that the patient can describe and perhaps in association with fever and hopefully supported by a urine sample.
Sometimes it can be difficult if you've got a patient who's had a stroke and can't talk to you, for instance, but I wouldn't assume that just because you've got E. coli in the urine that you've got a urinary tract infection requiring antibiotics.

Stephen uses passive voice when describing diagnostic practices (“you might,” “it does not mean anything”) indicating impersonality but uses first person pronoun “I” when emphasizing that he would not diagnose based on *E. coli* in urine, coming across as his personal perspective. Stephen shifts between reflecting on symptoms, acknowledging that identifying symptoms is difficult with older adult patients who cannot talk after stroke. Despite reflections, the narrative is coherent; yet, it does not achieve the usual resolution of restitution narrative, whereby biomedicine cures disease but ends up a narrative underlining refraining from making UTI diagnosis based on laboratory results even if coherence creates an affective sense of control.

Like the nurses' control narratives of UTI diagnosis, junior doctors' descriptions may resemble the stock responses discussed by Atkinson (1984), illustrating the unreflective sense of doing the right thing, even if the practices could fuel antibiotic overprescribing. However, Stephen's narrative illustrates the possibility of coherent counter-narratives to diagnosing UTIs based on bacteria identified in urine, which may be afforded by his senior position.

## Discussion

We contend that our findings related to chaos and control narrative interlacing our participants' accounts make conceptual, practical and methodological contributions to understanding diagnosis in hospitals, especially diagnosing UTIs in older adults.

Conceptually, our study addresses the contention between health research, which typically considers adherence to evidence, such as clinical guidelines, in medicine important, and medical sociologists, who have expressed concerns that evidence may lead to “cook book” medicine that is not sensitive to the complexities of clinical situations and patient experiences (Timmermans and Angell, 2001). Research has found that clinicians may use evidence differently, more in cook book fashion or critically reflecting on it (Timmermans and Angell, 2001), ignore guidance when it contradicts their “feel for the game” (Broom et al., 2014) and find a balance between flexibility and rigidity in using guidelines (Johannessen, 2017). These observations further relate to a classical discussion on how clinicians respond to uncertainty in a reflective way (Fox, 1980) or resorting to stock responses (Atkinson, 1984; Broom et al., 2014) or both (Timmermans and Angell, 2001).

Our chaos and control narratives share features of reflective and stock responses to uncertainty, respectively, but they also complicate them. Reflection has been discussed as an intellectual questioning attitude open to different perspectives and possible actions (Timmermans and Angell, 2001) or as an almost existential experience of self-doubt (Fox, 1980). Whilst chaos narratives have aspects of critical reflection, they typically did not indicate opting for different lines of action but a sense of being overwhelmed by contradictory experiences and events. Doubts about diagnosis were not only the purview of doctors but also underpinned the narratives of nurses and older adult patients and articulated their powerlessness. The control aspects of narratives resemble clinicians' adherence to customary mindlines or stock responses (Atkinson, 1984; Johannessen, 2017), but they also illustrated the affective sense of control and order afforded by following customary practices that comfortingly promised to restore health. However, control narratives also became intelligible in relation to experiences of chaos, highlighting how urinary dipsticks and antibiotics became means of bringing order to and thwarting the chaos of overwhelming evidence and illnesses. At the same time, being control could also sometimes articulate a different, non-dogmatic line of action.

Clinicians and patients articulated affective experiences of chaos or control in particular in relation to two aspects of diagnosing UTIs in older adults. Chaos and control became prominent when clinicians and older adult patients' described contradictory evidence, such as vague signs and symptoms of “being unwell,” and contradictory results of diagnostic tests, partly reflecting the tensions between the ostensibly objectively pathological evidence and the patient-centered subjective evidence of symptoms (Armstrong, 2011), confounded by new guidance. The other aspect provoking chaos and control narratives was aspects of caring for older adults, such as potential cognitive impairment, difficulties in understanding, and multiple illnesses and medications typical of old age. These issues of identifying and treating acute illnesses in old age boil down to the basic contradiction in hospital medicine identified by Strauss et al. (1987) that hospitals were originally geared toward treating acute illnesses and are poorly equipped to deal with chronic illnesses of old age they currently mostly cater for. The affective experiences of chaos and control were different ways of responding to the contradiction at the heart of acute model of hospital medicine, which led to the investigation of a suspected UTI in an older adult who was generally unwell.

The practical contribution of our study is the observation that the descriptions of how the diagnosis of a suspected UTI in an older adult proceeded was often very similar throughout our interviews and similar to quantitative and qualitative descriptions (Pallin et al., 2014; Eyer et al., 2016), the difference being the affective sense of doubt or certainty underpinning the accounts. Older adult patient views of UTI diagnosis have not, to our knowledge, been investigated, and our findings highlight that patients' experiences may articulate a sense of bewilderment with repeated UTI diagnoses and courses of antibiotics rather than pressuring for antibiotics, as indicated by clinician accounts (Charani et al., 2013; Eyer et al., 2016). Eventually the different affective experiences are likely to complicate communication and cooperation between staff groups and patients, an important component of suboptimal antibiotics prescribing (Lewis and Tully, 2009; Charani et al., 2013; Skodvin et al., 2017; Saukko et al., 2019). This is especially the case as junior doctors' and nurses' perspectives were often different, junior doctors doubting the diagnostic processes more often, perhaps due to a more reflexive occupational disposition (Fox, 1980; Timmermans and Angell, 2001) and higher awareness of new guidance. Efforts to reduce inappropriate antibiotic prescribing in hospitals have mostly focused on top-down education of clinicians (Davey et al., 2017). Whilst these interventions have been mostly effective (Davey et al., 2017) they focus on cognitive change and do not often involve patients. Our findings suggest that there is a rarely examined or acknowledged affective underlay that shapes clinicians' and patients' understandings and actions vis a vis diagnosis and antibiotic prescribing. To address this affective dimension would likely require a more conversational and cooperative approach to improving diagnosis and prescribing to enable the often unspoken insecurities and securities to be discussed.

Finally, our study also offers methodological insights on how to analyse the important affective dimension of experience through forms of narratives. The favored method for analyzing interviews in medical sociology is thematic analysis. Thematic analysis is flexible but focuses on *what* people tell rather than *how* they tell about their experiences. Our study offers ideas on how to analyse the way in which individuals position themselves in relation to unfolding events, whether the sequence of events is orderly and whether the story achieves resolution or closure. These elements highlight the not directly spoken way in which people position themselves as in charge of or at the mercy of events and whether they are indirectly doubting or certain about their or others' actions. Considering these often unspoken aspects of experience could shed new conceptual and practical light on why overdiagnosis and overprescribing happens.

## Data Availability Statement

The datasets generated for this study are available on request to the corresponding author.

## Ethics Statement

The studies involving human participants were reviewed and approved by Health Research Authority (IRAS 202255). The patients/participants provided their written informed consent to participate in this study.

## Author Contributions

ER and PS designed the study, contributed to analysis, and writing of the manuscript. PS led the qualitative data collection and analysis, and writing of the manuscript. All authors contributed to the article and approved the submitted version.

## Conflict of Interest

The authors declare that the research was conducted in the absence of any commercial or financial relationships that could be construed as a potential conflict of interest.
